# Developing a genetic approach to target cyanobacterial producers of heterocyte glycolipids in the environment

**DOI:** 10.3389/fmicb.2023.1257040

**Published:** 2023-09-27

**Authors:** Ruth Pérez Gallego, Nicole J. Bale, Jaap S. Sinninghe Damste, Laura Villanueva

**Affiliations:** ^1^Department of Marine Microbiology and Biogeochemistry (MMB), NIOZ Royal Netherlands Institute for Sea Research, Den Burg, Netherlands; ^2^Department of Earth Sciences, Faculty of Geosciences, Utrecht University, Utrecht, Netherlands

**Keywords:** heterocytous cyanobacteria, N_2_ fixation, heterocyte glycolipids, glycosyltransferase, lipid biosynthesis, polymerase chain reaction, primer design, environmental detection

## Abstract

Heterocytous cyanobacteria are important players in the carbon and nitrogen cycle. They can fix dinitrogen by using heterocytes, specialized cells containing the oxygen-sensitive nitrogenase enzyme surrounded by a thick polysaccharide and glycolipid layer which prevents oxygen diffusion and nitrogenase inactivation. Heterocyte glycolipids can be used to detect the presence of heterocytous cyanobacteria in present-day and past environments, providing insight into the functioning of the studied ecosystems. However, due to their good preservation throughout time, heterocyte glycolipids are not ideal to detect and study living communities, instead methods based on DNA are preferred. Currently cyanobacteria can be detected using untargeted genomic approaches such as metagenomics, or they can be specifically targeted by, for example, the use of primers that preferentially amplify their 16S rRNA gene or their *nifH* gene in the case of nitrogen fixing cyanobacteria. However, since not all cyanobacterial nitrogen fixers are heterocytous, there is currently no fast gene-based method to specifically detect and distinguish heterocytous cyanobacteria. Here, we developed a PCR-based method to specifically detect heterocytous cyanobacteria by designing primers targeting the gene (*hglT*) encoding the enzyme responsible for the last step in the biosynthesis of heterocyte glycolipid (i.e., a glycosyltransferase). We designed several primer sets using the publicly available sequences of 23 heterocytous cyanobacteria, after testing them on DNA extracts of 21 heterocyte-forming and 7 non-heterocyte forming freshwater cyanobacteria. The best primer set was chosen and successfully used to confirm the presence of heterocytous cyanobacteria in a marine environmental sample.

## Introduction

Cyanobacteria are cosmopolitan photosynthetic organisms with a central role in earth’s biogeochemical cycles. Besides their role as primary producers, fixing carbon through photosynthesis using only sunlight and CO_2_, a variety of cyanobacterial species are also capable of fixing atmospheric nitrogen (N_2_), which also confers them an important role in the nitrogen cycle (Zehr, 2011; Mishra et al., 2021).

Nitrogen fixation is catalyzed by the nitrogenase enzyme which is easily inactivated in presence of oxygen. Hence, cyanobacteria have developed two main strategies to protect the nitrogenase from the oxygen generated during photosynthesis. One strategy is to separate both processes in time; in most nitrogen-fixing unicellular cyanobacteria N_2_ fixation only occurs at night, when the photosynthetic machinery is not in use and oxygen is not being produced (Millineaux et al., 1981). Filamentous cyanobacteria developed an alternative strategy, i.e., the spatial separation of both processes. Heterocytous cyanobacteria can develop heterocytes, a type of specialized cell where, when in absence of combined nitrogen, nitrogen fixation takes place (Klugkist and Haaker, 1984; Knutson et al., 2018). Heterocytes protect the nitrogenase enzyme from oxygen using several strategies such as (1) providing spatial separation of nitrogen and carbon fixation by confining nitrogen fixation to specialized cells surrounded by a thick-walled outer membrane composed of polysaccharide (heterocyte envelope polysaccharide, HEP) and an inner layer composed of heterocyte glycolipids (HGs) (Haselkorn, 1978), (2) inactivation of photosystem II in heterocytes to avoid production of oxygen (Tel-Or et al., 1997), and (3) enhanced respiration inside the heterocytes to consume internal oxygen (Wolk et al., 1994).

Currently, the presence of cyanobacteria in the environment can be confirmed by using cyanobacteria-specific 16S rRNA gene primers (Nübel et al., 1997), and the presence of dinitrogen-fixing cyanobacteria by applying primers targeting *nifH* (Olson et al., 1998), a key gene involved in dinitrogen fixation. However, there are no screening techniques based on genetic methods allowing the detection of heterocytous cyanobacteria in the environment, which could be used to evaluate the potential for cyanobacterial N_2_ fixation. Thus far, we can only determine the presence of heterocytous cyanobacteria using time and resource intensive methods such as microscopy and lipid analysis, or by using untargeted metagenomic methods. Taxonomic classification of cyanobacteria via microscopic analysis requires expertise and relies solely in morphological traits (Castenholz et al., 2001).

Cyanobacterial taxonomy is complex and has undergone extensive restructuring and revisions in recent years. It has transformed from being a hierarchical system that simply placed morphologically similar taxa into a classification system based on evolutionary relationships. Most of the studies mentioned in this work used the classification system proposed by Rippka et al. (1979), later adopted as the classification standard in the Bergey’s Manual of Systematic Bacteriology (Castenholz et al., 2001). This classification method recognized five subsections or orders, which in turn contained “families,” divided mainly according to their morphology. Heterocytous cyanobacteria occur only in subsections IV (*Nostocales*) and V (*Stigonematales*). Although this nomenclature did not reflect evolutionary relationships, it was adopted as convenient and temporary method to classify cyanobacterial strains (Castenholz et al., 2001). However, a recent revision of cyanobacterial taxonomy by Komarek et al. (2014) proposed a system that left aside classification based solely on morphology and focused on a polyphasic approach focusing on gene phylogeny (e.g., 16S rRNA) to achieve monophyletic clusters representing “orders.”

**Figure 1:**
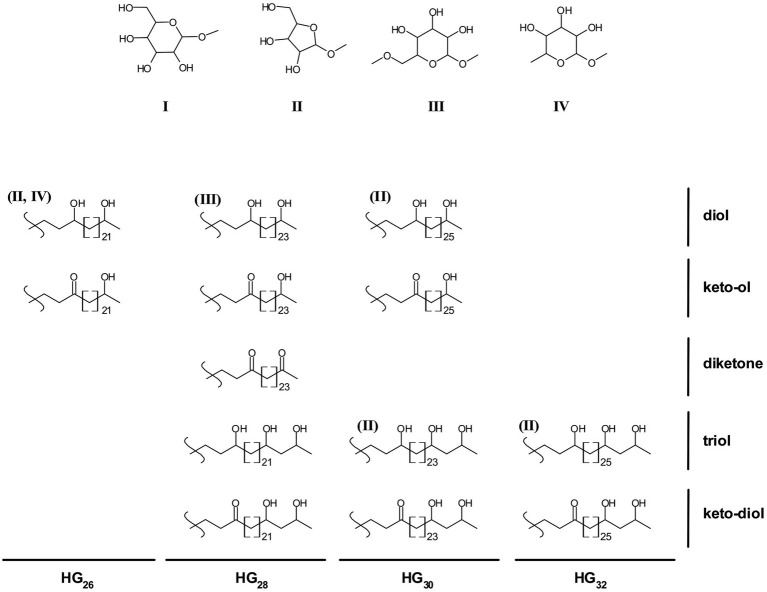
Chemical structures of heterocyte glycolipids found in cyanobacteria. I-IV depict the potential HG headgroups: hexose (I), pentose (II), methyl hexose (III), deoxyhexose (IV). All depicted HG structures usually contain a hexose headgroup (I), some HGs have also been observed containing alternative headgroups (shown in brackets above the HG structure).

The chemical structure of the HGs and their distribution (Figure 1) has chemotaxonomical value and can potentially also be used to distinguish between cyanobacteria of different orders and families (Table 1; Supplementary Table S1; Gambacorta et al., 1998; Bauersachs et al., 2009a, 2013). For example, hexose-sugar HGs composed of C_26_ (HG_26_) diol and keto-ol are mostly present in species of the *Nostocaceae* family (*Nostocaceae* and *Aphanizomenonaceae* orders according to the new 16S rRNA-based classification; Gambacorta et al., 1998; Bauersachs et al., 2009a; Komarek et al., 2014; Table 1; Supplementary Table S1), while hexose C_28_ HGs (HG_28_) containing three functional groups (keto-diol and triol) are predominant in members of the *Rivulariaceae* (*Calotrichaceae*) family. Likewise, hexose C_30_ HG (HG_30_) triols and keto-diols are characteristic of members of the *Scytonemataceae* (*Scytonemataceae* and *Tolypothrichaceae*) family, while members of the *Stigonemataceae* (*Stigonemataceae*, *Chlorogloeopsidaceae* and *Hapalosiphonaceae*) mostly synthesize hexose C_32_ HGs (HG_32_) with three functional groups (Gambacorta et al., 1998). However, there are some exceptions to these general trends. For example, the *Nostocaceae* (*Nostocaceae* and *Aphanizomenonaceae*) family mostly produces hexose HG_26_ diol and keto-ol, but there are members of this family that also produce hexose HG_28_ triol and keto-diol (Supplementary Table S1). Although they are usually produced in small amounts, in some cases they are synthesized in larger amounts, such as in *Aphanizomenon* sp. TR83 (12% of the total HGs) and in *Dolichospermum* sp. BIR169 (33%) (Bauersachs et al., 2017; Supplementary Table S1). Moreover, *Stigonema ocellatum* SAG 48.90, a strain belonging to the *Stigonematales* order (*Stigonemataceae*), was recently shown to produce mostly hexose HG_28_ keto-diol (67%) and triol (27%), HGs thought to be characteristic of the *Rivulariaceae* (*Calotrichaceae*) family (Supplementary Table S1; Bauersachs et al., 2019).

**Table 1:** Summary of HGs produced by different cyanobacterial families according to the classification described in Komarek et al. (2014) and to the nomenclature in the original studies.*,**

Family in original study	Family as per Komarek et al. (2014)	HG_26_ diol keto-ol and diol	HG_28_ keto-ol and diol	HG_28_ keto-diol and triol	HG_30_ keto-ol and diol	HG_30_ keto-diol and triol	HG_32_ keto-diol and triol
*Nostocaceae^abc^*	*Aphanizomenonaceae*	++	(++ − tr.)	(+ − tr.)			
*Nostocaceae^abdefg^*	*Nostocaceae*	++	(+ − tr.)				
*Rivulariaceae^abehi^*	*Calotrichaceae*		(+ − tr.)	++			
*Rivulariaceae^h^*	*Fortieaceae*		++		tr.	tr.	
*Scytonemataceae^h^*	*Tolypothrichaceae*		++			+	
*Scytonemataceae^hi^*	*Scytonemataceae*					++	
*Stigonemataceae^i^*	*Stigonemataceae*		+	++		tr.	
*Stigonemataceae^h^*	*Chlorogloeopsidaceae*						++
*Stigonemataceae^hij^*	*Hapalosiphonaceae*						++
*Fischerellaceae^j^*	*Hapalosiphonaceae*						++
*Nostochopsidaceae^j^*	*Hapalosiphonaceae*						++

++ dominant, + present, tr. trace amounts.

*^a^*Bauersachs et al. (2009b), *^b^*Wörmer et al. (2012), *^c^*Bauersachs et al. (2014), *^d^*Bauersachs et al. (2010), *^e^*Bauersachs et al. (2009a), *^f^*Gambacorta et al. (1996), *^g^*Schouten et al. (2013), *^h^*Gambacorta et al. (1998), *^i^*Bauersachs et al. (2019), *^j^*Bauersachs et al. (2014).

*In this work when discussing strains analyzed in previous studies in the main text, we will use the taxonomic classification as described in the original study (first column of this table), as well as their family-level classification as per described in Komarek et al. (2014) (second column of this table), the latter will be shown in between brackets.

**Values without brackets indicate HGs present in all strains of the indicated family, while values in brackets indicate HGs present only in some strains. Hyphen symbol (−) indicates a range of abundances.

Additionally, the type of headgroup of HGs (Figure 1) can also be an indicator of the lifestyle and habitat of the producing heterocytous cyanobacterial strain. Most HG-producing cyanobacteria biosynthesize HGs with a hexose (C_6_) as headgroup, however some cyanobacterial species also produce HGs with alternative headgroups such as pentoses (C_5_), deoxyhexoses (C_6_) or methyl-hexoses (C_6_) (Schouten et al., 2013; Bale et al., 2018a; Figure 1; Supplementary Table S1). Most of the heterocytous cyanobacterial strains studied to date that synthesize HGs with a hexose headgroup are free-living and inhabit freshwater and terrestrial environments, with a few exceptions, i.e., *Nostoc muscorum* UTEX 1933 and *Calothrix* sp. UTEX 2589 synthesize HG_26_ and HG_28_ with hexose as headgroup but were enriched from marine environments (Schouten et al., 2013). In contrast, heterocytous cyanobacteria that are symbionts of marine diatoms, synthesize HG_30_ and HG_32_ HGs containing a pentose headgroup, and were, consequently, proposed as biomarkers for tracing endosymbiotic heterocytous cyanobacteria in present and past marine environments (Schouten et al., 2013; Bale et al., 2018b).

**Figure 2:**
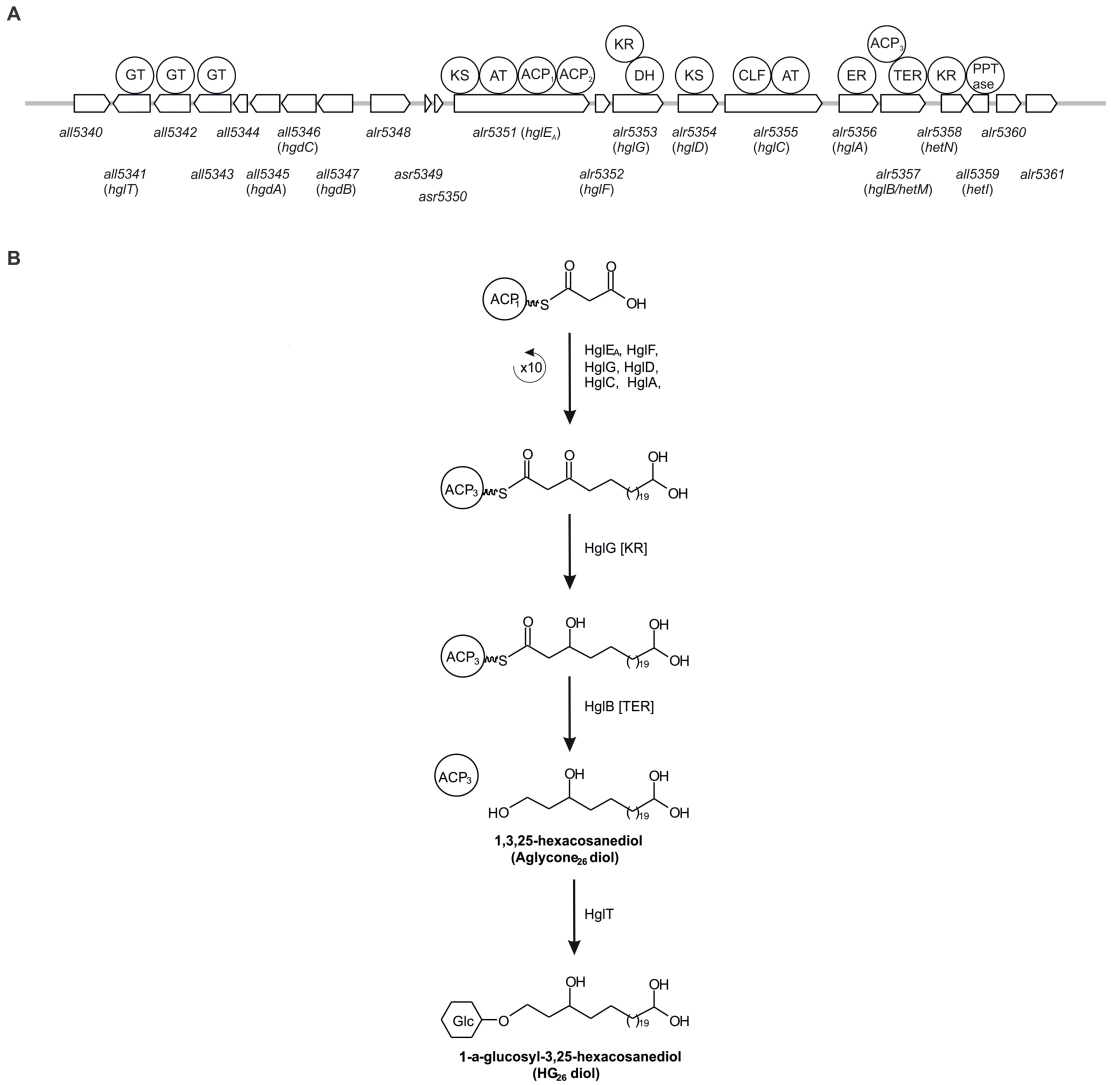
Schematic representation of the genes involved in HG biosynthesis in Anabaena sp. PCC 7120 **(A)** and simplified representation of the biosynthesis pathway of HG26 diols **(B)**. ACP, acyl carrier protein; AT, acyl transferase; CLF, chain length factor; DH, dehydrase; ER, enoyl reductase; GT, Glycosyltransferase; KR, ketoacyl reductase; KS, β-ketoacyl synthase; TER, thioester reductase.

Due to their potential preservation over geological time scales, HGs have also been used as biomarkers to study the occurrence of nitrogen fixation in ancient sediments (Bauersachs et al., 2010). In addition, changes in the abundances of HG_26_ diols and keto-ols can be used to reconstruct surface water temperatures in freshwater environments using the HDI_26_ index (Bauersachs et al., 2015). However, our current understanding of HG production among heterocytous cyanobacteria is restricted, because only a limited amount of HG-producing cyanobacterial cultures has been examined. A solution would be to study the heterocytous cyanobacterial community occurring in the environment using both approaches, i.e., HG and genetic analyses. One could use untargeted genetic approaches such as 16S rRNA and *nifH* gene analysis using primers that specially target cyanobacteria. However, these approaches might not be specific enough, since they will target all cyanobacteria and N_2_ fixing cyanobacteria (among other N_2_ fixing organisms; Olson et al., 1998). Hence, if the species of interest are sparse, there is the risk of overseeing them and detecting only the more abundant organisms. Therefore, ideally, the genetic analysis should target genes exclusive to heterocytous cyanobacteria such as those involved in HG biosynthesis (Figure 2).

In this study, we developed a specific PCR-based molecular approach designed to specifically target heterocytous cyanobacteria. To this end, we developed primers targeting *all5341* (also known as *hglT*), a gene essential for HG biosynthesis but not essential for survival, encoding the glycotransferase involved in the last step of the synthesis of HGs in *Anabaena* sp. PCC 7120 (Awai and Wolk, 2007; Halimatul et al., 2014) using genomes available from public databases. We validated them using 28 freshwater cyanobacterial cultures, optimized the PCR conditions, and tested them in the environment.

## Materials and methods

### Nucleic acids extraction

#### Cyanobacterial cultures

Cyanobacterial biomass from strains listed in Table 2 was obtained from frozen (−80°C) pellets stored in the former Culture Collection Yerseke (CCY) and starter cultures provided by the Pasteur Culture Collection (PCC). Except *Chysosporum ovalisporum* strains UAM290 and UAM292, kindly provided by Dr. Samuel Cirés and *Anabaena* sp. PCC 7120, kindly provided by Dr. Koichiro Awai. Genomic DNA was extracted using the DNeasy PowerSoil kit (Qiagen, Hilden, Germany) according to the manufacturer’s instructions with minor modifications, namely: samples were disrupted using a mixture of 0.2 mL 0.5 mm diameter and 0.2 mL 0.1 mm diameter glass beads (BioSpec Products, Bartlesville, OK, USA) on the bead mill homogenizer (Bead Ruptor Elite, Omni International, Kennesaw, GA, USA) running twice for 10 s at a speed of 3.55 m s^−1^ with a 30 s dwell. Concentration of DNA extracts was analyzed using a Nanodrop ND-1000 Spectrophotometer (NanoDrop Technologies Inc. Wilmington, DE, USA).

**Table 2:** Summary of the strains used in this study to test the designed primers.

Heterocytous	
*From Culture Collection Yerseke (CCY)*	
CCY 0012	*Nostoc*
CCY 0017	*Anabaena*
CCY 0018	*Calothrix*
CCY 0103	*Nodularia chucula*
CCY 0202	*Calothrix*
CCY 9402-a	*Anabaena*
CCY 9613	*Anabaena*
CCY 9614	*Anabaena*
CCY 9626*	*Nostoc*
CCY 9910	*Anabaena*
CCY 9921	*Anabaena cylindrica*
CCY 9922	*Anabaena variabilis*
CCY 9923	*Calothrix*
CCY 9924	*Chlorogloeopsis* sp.
CCY 9925	*Nostoc*
CCY 9926	*Nostoc*
*From Pasteur Culture Collection (PCC)*	
PCC 7101	*Tolypothrix tenuis*
PCC 10023	*Scytonema* sp.
*Other sources*	
PCC 7120*	*Anabaena* sp.
UAM 290	*Chrysosporum ovalisporum*
UAM 292	*Chrysosporum ovalisporum*
Non heterocytous	
*From Culture Collection Yerseke (CCY)*	
CCY 9627	*Leptolyngbya*
CCY 9409	*Lyngbya* sp.
CCY 9703	*Pseudanabaena*
CCY 0011	*Synechococcus*
CCY 9201	*Synechococcus*
CCY 9503	*Synechococcus*
CCY 9506	*Synechococcus*

*Indicates the same strain kept in two different culture collections under different identification numbers.

#### Environmental samples

DNA from a microbial mat collected from Schiermonnikoog station 3 (29′ 28.3626″, 6° 8′ 20.9646″) on 10th April (Fan et al., 2015), was extracted from freeze dried homogenized sample using the same extraction kit and sample homogenization protocol as described above for cyanobacterial cultures. A DNA extract of the seaweed *Sargassum fluitans* was kindly provided by Tom Theirlynck and Linda Amaral-Zettler. The *S. fluitans* sample was collected from the Central Atlantic Ocean (station 24: 8° 26′ 6.1548″, −49° 44′ 27.2142″) during cruise 64PE455 on board of the *R/V* Pelagia during July–August 2019 as described in Theirlynck et al. (2023). DNA extraction of *S. fluitans* sample number 38 is described in Theirlynck et al. (2023).

### Lipid extraction and analysis

Lipids present in the microbial mat sample were extracted from freeze dried aliquots using a modified Bligh-Dyer procedure as described by Bauersachs et al. (2011 and references within). The extracted intact polar lipids were analyzed by high performance liquid chromatography coupled to electrospray ionization tandem mass spectrometry (HPLC/ESI-MS^2^) as described in Bauersachs et al. (2009b).

### Polymerase chain reaction (PCR) and sequencing

#### Validation of the DNA extracted from cyanobacterial cultures

To ensure that the DNA extracted from cyanobacterial cultures was of good quality and suitable for our study, we performed a PCR using universal primers 515F-Y and 806RB targeting the 16S rRNA gene (Supplementary Table S2; Walters et al., 2015), resulting in the expected ~300 bp band. All PCR reaction mixes and cycles used in this study were as described in Besseling et al. (2018) with minor modifications, i.e., PCR annealing temperature was 50°C, extension time was 30″ and it was repeated for 30 cycles (hereafter these specifications will be shown within parenthesis in the main text).

#### Amplification and sequencing of *hglT*

Upon optimisation of the PCR conditions (see Results), amplification of *hglT* using the primers designed in this study was carried out using an annealing temperature 56.2°C, 1′20″ extension time, for 30 cycles, and an equimolar mixture of primers, for example Rv1 refers to an equimolar mixture of Rv1_G1, Rv1_G2, Rv1_G3, Rv1_G4 and Rv1_G5.

Upon amplification of *hglT* with primer mixes Fw1 mixB and Rv1, the presence of a single or multiple PCR products was analyzed via electrophoresis on an agarose gel (1%) stained with ethidium bromide or GelRed® (Biotium, Hayward, CA, USA), using SmartLadder (Eurogentec, Seraing, Belgium) as molecular weight marker. If only one band was observed the PCR product was purified using a PCR purification kit (QIAquick PCR Purification kit, Qiagen, Hilden, Germany). If more than one band was observed, then the PCR product was run on 1% or 1.5% agarose gels stained with SYBR Safe® (ThermoFisher Scientific, USA), bands were then excised and purified using the QIAquick gel extraction kit (Qiagen, Hilden, Germany). To confirm the purity of the purified bands a small aliquot was run again on an agarose gel (1%) as described above. If the amount of the PCR product was not sufficient for direct sequencing, the amount was increased via concentration of the product of several PCR reactions using a PCR purification kit (QIAquick PCR Purification kit, Qiagen, Hilden, Germany). Purified PCR products were sequenced via Sanger sequencing at Macrogen Europe (Amsterdam, The Netherlands) using primer mixes Fw1 mixB and Rv1.

If after concentration the amount of PCR product was still not sufficient for direct sequencing, it was then cloned into the pCR™4-TOPO™ vector (Invitrogen, Carlsbad, CA, USA) using the TOPO-TA cloning kit for sequencing (Invitrogen, Carlsbad, CA, USA) according to the manufacturer’s instructions. The ligation product was subsequently transformed into TOP10 competent cells (Invitrogen, Carlsbad, CA, USA) and plated in LB plates containing ampicillin (100 μg/mL), kanamycin (20 μg/mL), IPTG (0.1 mM) (UltraPure™ IPTG, Invitrogen Europe, manufactured in Italy) and X-Gal (0,2 mg/mL) (Thermo Scientific, Vilnius, Lithuania). The resulting white colonies were collected using a sterile toothpick, dissolved in 50 μL TE 1x buffer and lysed by incubation at 95°C for 10 min. Upon centrifugation at 2000 rpm for 1 min, 1 μL supernatant was used as template in 25 μL PCR reactions using M13 primers (Supplementary Table S2) and the PCR reaction mix and amplification program described above. PCR products were sent to Macrogen Europe for PCR purification and Sanger sequencing using primers T3 and T7 (Supplementary Table S2).

#### Amplification and sequencing of 16S rRNA

16S rRNA V4-V5 regions from DNA present in the microbial mat sample was amplified using barcoded primers 515F-Y and 926Rbc (Parada et al., 2016) via PCR reaction using Phusion Taq Polymerase (Thermo Fischer Scientific). And the following PCR program: 30″ at 98°C, 30 cycles of 10″ at 98°C, 20″ at 50°C, 30″ at 72°C; final extension at 72°C for 7′ and storage at 4°C. Samples were amplified in triplicate and pooled together after positive confirmation via electrophoresis on an agarose gel (2%). Positive controls (*E. coli* lysate) and negative controls (PCR water without DNA) were included. Concentration of each PCR product was quantified on an agarose gel (2%) using a quantification standard. PCR products from all samples were pooled in equimolar concentrations, ran on an agarose gel (2%) and the resulting ~400 bp band was cut off the gel and purified using QIAquick gel extraction kit (Qiagen, Hilden, Germany) according to the manufacturer’s instructions. Sequencing was performed by Macrogen Europe (Amsterdam, The Netherlands) using an Illumina MiSeq 2 × 300 bp V3 kit (Illumina, CA, USA). Amplification and analysis of 16S rRNA gene of the *S. fluitans* sample is described in Theirlynck et al. (2023).

### Bioinformatic analysis

#### Search of HglT homologs and primer design

Homologs of the protein HglT of *Anabaena* sp. PCC 7120, encoded by gene *all5351* (IMG gene ID 637235753 hereafter, *hglT*) were found via BLASTx (translated nucleotide sequence searched against protein sequences, accessed on May 2020) (Altschul et al., 1990; Camacho et al., 2009) against 23 genomes (Supplementary Table S3 and Supplementary Figure S1). The resulting gene sequences (Supplementary Table S4) were aligned in MEGA (v11.0.8) using the in-built MUSCLE (Edgar, 2004) sequence aligner with default parameters [gap open penalties: −400, gap extend penalties: 0, max iterations: 16, Cluster Method: UPGMA, Min Diag Length (Lambda): 24]. To select suitable regions for the design of our primers we used the following guidelines: (1) Binding region should be 17–22 base pairs (bp) long; (2) Binding region Tm’s should be 50–65°C; (3) Binding region should have moderate GC content (<50%); (4) Repetitive poly-N regions should be avoided; (5) Regions prone to secondary structure formation (hairpins, self-dimerization or primer dimer), such as regions containing complementary sequences (palindromes) within themselves should be avoided; (6) Primer pairs should have similar Tm’s (±4°C); and (7) Distance between the two binding regions (Fw and Rv) should be as large as possible. The regions comprised between the base pairs 112..131 and 299..320, were selected as suitable regions for the design of forward primers, and regions 949..968 and 973..989 were selected as targets for the design of reverse primers.

Primers were designed according to the general primer design guidelines described by Abd-Elsalam (2003), which we applied by setting the following rules: (1) length of the primer should be between 17 and 22 bp (2) degenerate nucleotides should not be consecutive (3) degenerate nucleotides should be as far apart from each other as possible (4) each primer sequence should have the least number of degenerate nucleotides possible but no more than four. Primer properties such as melting temperature (Tm) and percentage of GC content were also taken into consideration, aiming to design primers with similar Tm and moderate GC content (<50%). The potential of each primer to create hairpins and primer dimers due to potential self-annealing was also checked by using OligoCalc (Kibbe, 2007; see Supplementary Table S5). All primers were synthesized by Integrated DNA Technologies (IDT, Leuven, Belgium).

#### Analysis of *hglT* amplicons

Nucleotide sequences obtained via Sanger sequencing were processed using Geneious Prime (v 2023.0.4). Sequences were trimmed using an error probability limit of 0.01. Potential heterozygous bases in single reads were identified using a 50% peak similarity cutoff, peak detection height was set at 10%. When available, the consensus sequence was obtained by aligning forward and reverse reads with Geneious assembler using the highest sensitivity settings. When appropriate, sequences belonging to the pCR™4-TOPO™ vector (Invitrogen, Carlsbad, CA, USA) were identified and removed.

To determine their origin, the resulting sequences were queried using Geneious built-in blastN (BLAST+ v 2.12.0, accessed in March 2023) (Altschul et al., 1990; Camacho et al., 2009) with default parameters (low complexity filter, mask for lookup table, max e-value: 0.05, word size: 11, open gap cost: 5, extend gap cost: 2, scoring match: 2, scoring mismatch: 2–3).

To generate the phylogenetic trees sequences were aligned using MAFFT (v7.407) with L-INS-i iterative refinement method (Katoh and Standley, 2013) and poorly aligned regions were removed using trimAl (Capella-Gutiérrez et al., 2009). Phylogenetic trees were built using IQ-tree (v1.6.7) and its in-built nucleotide substitution model finder (Kalyaanamoorthy et al., 2017), using 1,000 replicates to perform SH-like approximate likelihood ratio test (SH-aLRT) (Guindon et al., 2010) and 1,000 bootstrap replicates. Phylogenetic trees were visualized using iTOL (v6.7.4) (Letunic and Bork, 2021).

#### Analysis of 16S rRNA amplicons

The resulting amplicon sequencing data was processed using the Cascabel pipeline (Abdala Asbun et al., 2020). Within the Cascabel pipeline raw reads were filtered, trimmed and ASVs identified using the dada2 R package (v 1.19.1) (Callahan et al., 2016). Taxonomy was assigned using RDP (Wang et al., 2007) classifier within dada2, using SILVA SSU database 138 Ref NR 99 (Quast et al., 2013) and setting the minimum bootstrapping support required to return a taxonomic classification to 45% (minBoot = 45). Resulting taxa with a minimum count of two observations were summarized using QIIME (using summarize_taxa.py v 1.9.1 and filter_otus_from_otu_table.py v 1.9.1) (Caporaso et al., 2010) and BIOM tables were converted using BIOM tool (v 2.1.6) (McDonald et al., 2012). Processing and analysis of 16S rRNA amplicon sequencing data of the *S. fluitans* sample is described in Theirlynck et al. (2023).

## Results and discussion

### Assessing the potential of *hglT* as phylogenetic marker gene

**Figure 3:**
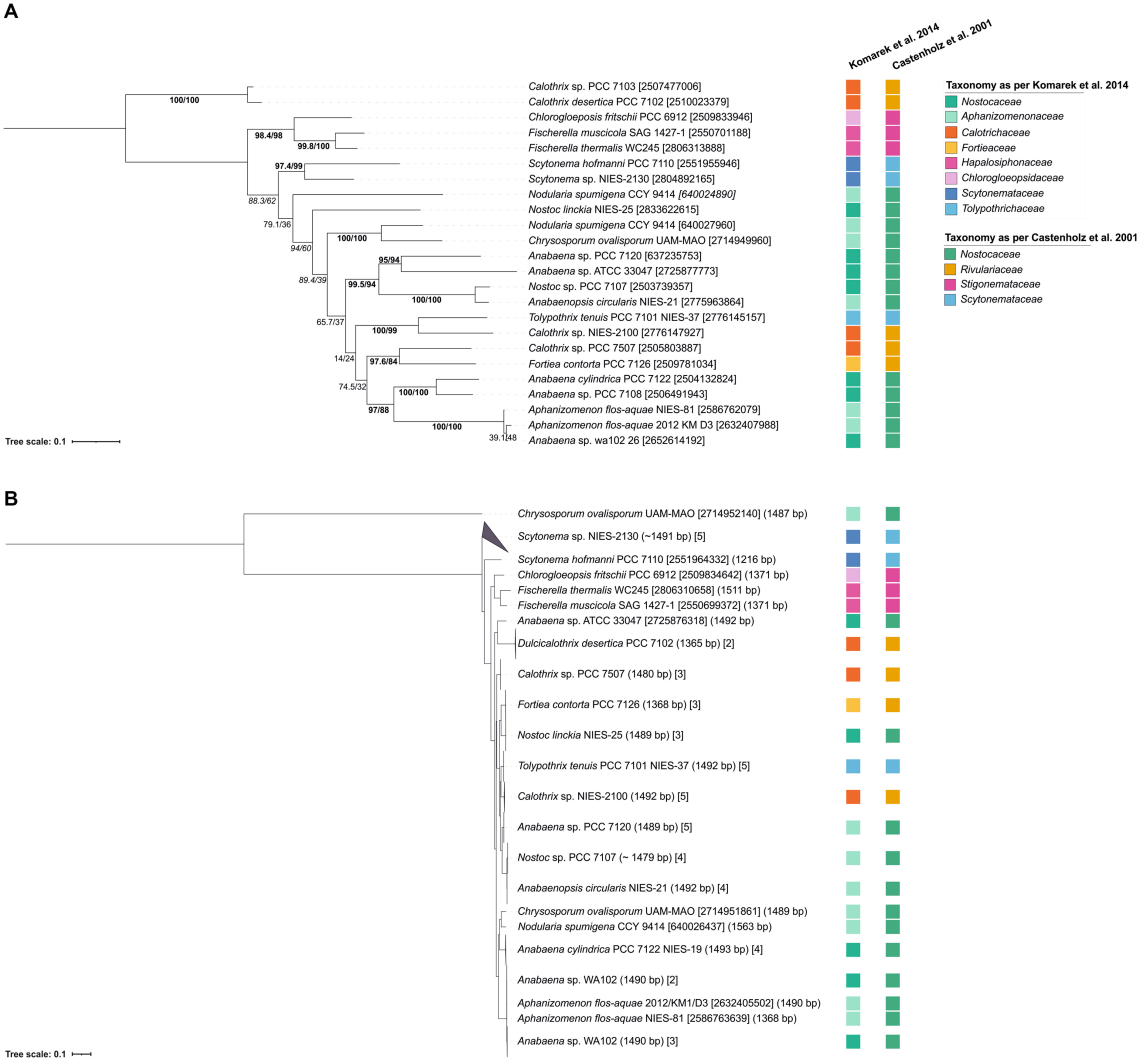
Tree containing **(A)** *hglT* gene sequences and **(B)** 16S rRNA sequences from selected heterocytous cyanobacteria (Supplementary Table S6). **(A)** *hglT* sequences (gene number shown in between brackets) (Supplementary Table S4) were obtained from selected genomes of heterocytous cyanobacteria (Supplementary Table S3). For each genome, we chose the sequence with the highest percentage of homology and highest bit-score (Supplementary Table S4). For the *Nodularia spumigena* CCY 9414 genome, two sequences with similar bit-score (439 and 420) and homologies of 78 and 64%, were included. **(B)** 16S rRNA gene sequences were obtained from IMG (Supplementary Table S6), numbers between parentheses indicate the gene length, while numbers between brackets indicate either the gene id (10 digit numbers) or the number of gene sequences included in the branch. In tree **(A)** values on branches indicate the SH-aLRT (left side of the slash) and the standard non-parametric bootstrap supports (right side of the slash). High SH-alrt and bootstrap supports (≥85 and ≥ 70, respectively) (Hillis and Bull, 1993; Anisimova et al., 2011) are shown in bold, when only SH-alrt support is high, values are shown in italics. Bootstrap values of tree **(B)** are shown in Supplementary Figure 2. Scale bar represents the mean number of substitutions per site.

To assess the phylogenetic potential of *hglT*, we generated a phylogenetic tree using complete *hglT* sequences from genomes available on the Integrated Microbial Genomes & Microbiomes system (IMG/M^1^) (Chen et al., 2021). We searched for homologs of the protein HglT of *Anabaena* sp. PCC 7120, encoded by gene *all5351* (IMG gene ID 637235753 hereafter, *hglT*) against 23 genomes (Supplementary Table S3) using BLASTx (translated nucleotide sequence searched against protein sequences), accessed on May 2020 (Altschul et al., 1990; Camacho et al., 2009). These included the genomes of 11 strains known to produce HGs, and 12 genomes of strains closely related to strains known to produce HGs. For example, *Chrysosporum ovalisporum* UAM 290 has been described to synthesize HGs (Wörmer et al., 2012) but there is no genome available for this strain, so we chose the genome of *Chrysosporum ovalisporum* UAM-MAO. In the BLASTx search, we selected for each genome, the protein sequence with the highest percentage of homology (average 75%; n = 23) and highest bit-score (average 421, *n* = 23) (Supplementary Table S4). These sequences were used to build the *hglT* phylogenetic tree (Figure 3A).

To ensure that the *hglT* gene tree provides enough resolution, we compared it to a 16S rRNA tree generated using sequences obtained from the same (or closely related) genomes as *hglT* (Figure 3B; Supplementary Figure S2; Supplementary Table S6). Cyanobacteria can possess multiple copies of the 16S rRNA gene, oftentimes also of different sequence length. Thus, to avoid potential misalignments caused by large differences in sequence length we only included sequences which were at least 1,000 bp long. Figure 3B reveals that, in most cases, the 16S rRNA sequences of the examined cyanobacteria cluster together, with a few exceptions, such as the two sequences of *Crysosporum ovalisporum* UAM-MAO.

The results from the *hglT* and 16S rRNA tree do not always show the same evolutionary history for a given genome. However, both trees agree with observations of previous studies, such as the polyphyletic origin of nearly all cyanobacterial families present in the trees, confirming previous reports in literature about the polyphyletic origin of *Nostoc*, *Anabaena*, *Calothrix*, *Tolypothrix* and *Scytonema* (Komárek et al., 2013; Komarek et al., 2014; Berrendero Gómez et al., 2016 and references cited therein), and the monophyletic origin of the *Stigonemataceae* family (*Chlorogloeopsidaceae* and *Hapalosiphonaceae*) (Castenholz et al., 2001 and references within). In general, it can be concluded that the *hglT* tree provides enough resolution to use this gene as a marker for HG-producing cyanobacteria.

### Primer design and validation in pure cultures

**Figure 4:**
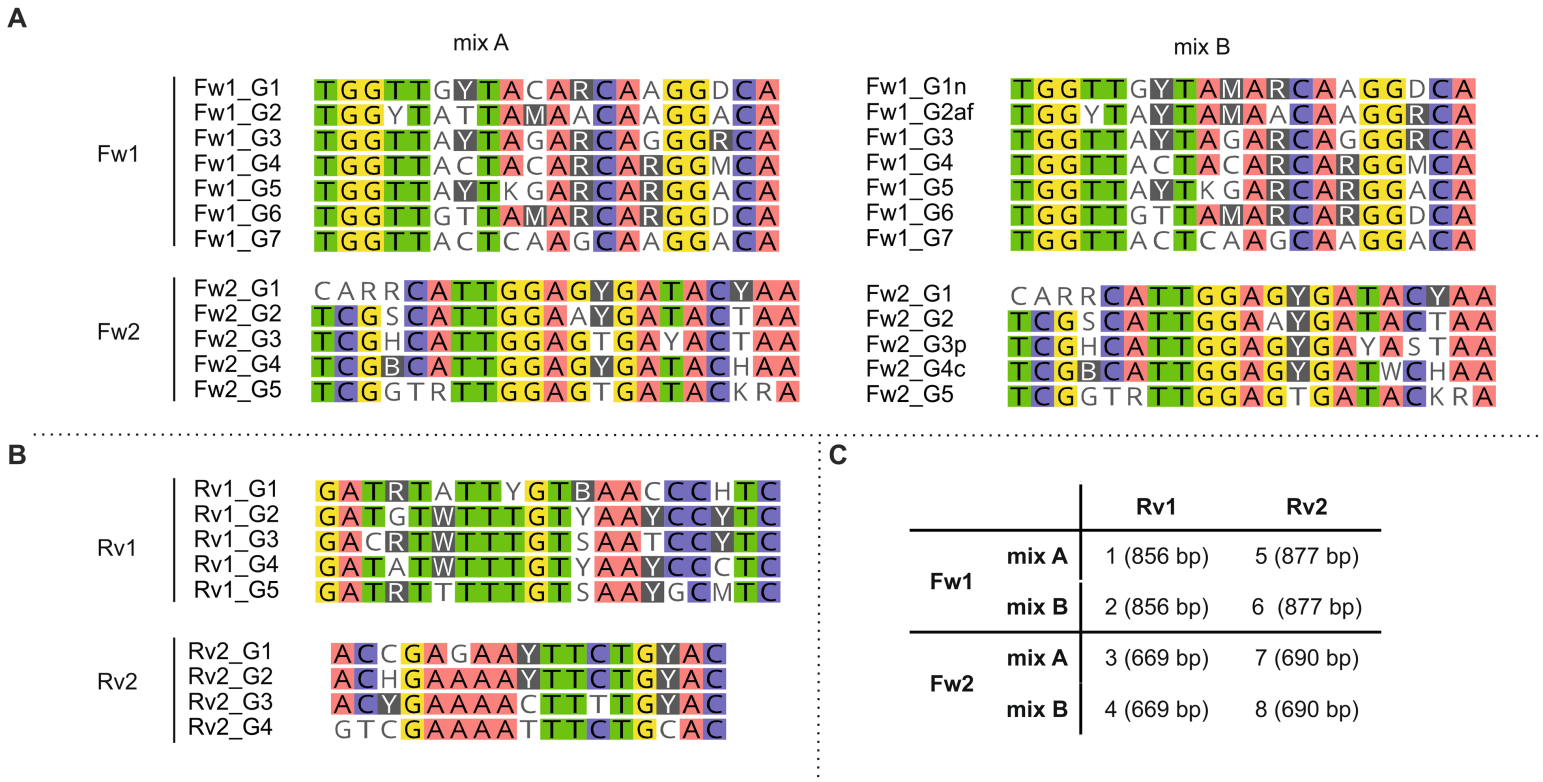
Primer sequences included in each forward **(A)** and reverse **(B)** primer mix. **(C)** Primer mixes included in primer sets 1–8 and in brackets the expected size of the resulting PCR products. Background colors show bases conserved (threshold: 75%) across the primer sequences. Primers were designed to target the most conserved gene regions.

To design primers targeting *hglT*, we used all sequences used to generate the *hglT* tree (Figure 4; Supplementary Table S4), aligned them in MEGA (v 11.0.8) using the in-built MUSCLE (Edgar, 2004) sequence aligner with default parameters, and manually identified regions suitable as primer targets (see Supplementary Figure S1 for details).

To be able to target the largest number of cyanobacterial species, while still following the rules considered for the design of the degenerate primers (i.e., only a maximum of four degenerate nucleotides should be included; see Material and Methods for details), we decided to design several primers targeting the same binding region but containing different combinations of degenerate bases (Figure 4; Supplementary Tables S2, S5), and use them in equimolar amounts in the PCR reaction. For some primers (Fw1_G1, Fw1_G2, Fw2_G3 and Fw2_G4), we also designed an alternative variant (Fw1_G1n, Fw1_G2af, Fw2_G3p and Fw2_G4c, respectively) (Supplementary Table S5) containing one additional degenerate nucleotide that would allow to increase the number of targeted strains (Supplementary Table S7). For example, Fw1_G1n contains 4 degenerate bases and targets 8 of the 24 strains, while Fw1_G1 contains 3 degenerate bases and targets only 6 strains. Both primer variants were tested in parallel and in combination with primers targeting the same region for which only one variant was designed (i.e., Fw1_G3 to Fw1_G7). For example, primer variants with the least number of degenerations (i.e., Fw1_G1 and Fw1_G2) were tested in combination with Fw1_G3 to Fw1_G7 (hereafter referred to as “mix A”), and primer variants with the greatest number of degenerations (i.e., Fw1_G1n and Fw1_G2af) were also tested in combination with Fw1_G3 to Fw1_G7 (hereafter referred to as “mix B”) (Figure 4).

The designed primers were tested using DNA extracted from 21 heterocyte-forming cyanobacterial strains (Table 2; Supplementary Table S8). Next, in order to develop the best suited primer set and its optimal annealing temperature, we performed a PCR with an annealing temperature gradient (52–63°C annealing temperature, 1′20″ extension time, 30 cycles) on two selected strains (*Calothrix* CCY 9923 and *Chlorogloeopsis* sp. CCY 9924) using all possible primer combinations (Supplementary Figure S3). All primer combinations successfully targeted the selected strains, and primer combinations containing a larger number of degenerate bases (mix B) did not show more unspecific bands than those containing the more conservative primers (mix A) (Supplementary Figure S3). Consequently, we decided to only use primer mix B to be able to target the largest number of cyanobacterial strains, and thus reduce the total number of primer combinations from eight to four. For strain *Calothrix* CCY 9923 primer combinations containing Fw1 primers resulted in an additional smaller (~400 bp) and unexpected band in the agarose gel when using an annealing temperature of 56.2°C or lower (Supplementary Figure S3). However, in some cases using an annealing temperature higher than 56.2°C resulted in much lower concentrations of PCR product, as was observed, for example, when primers Fw1 mixB and Rv2 and an annealing temperature of 58.2°C were used on *Chlorogloeopsis* sp. CCY 9924 (Supplementary Figure S3). Hence, despite the appearance of the unspecific band, we chose in favor of higher PCR product concentrations and chose to use an annealing temperature of 56.2°C in all subsequent PCR reactions. Next, we checked which of the four remaining primer pairs could amplify the *hglT* gene from the largest number of strains (Supplementary Figure S4). Primer pair 2 (Fw1 mix B + Rv1) successfully generated a PCR product of the expected size (~850 bp) for most of the tested strains (Supplementary Figure S4) and was selected as the best primer pair to potentially detect C_6_ HG producers in the environment.

To check the specificity of this primer pair (Fw1 mix B + Rv1) we also tested it in non-heterocyte forming cyanobacterial strains (Table 2; Supplementary Table S8). In four out of seven of these strains (*Synechococcus* strains CCY 0011, CCY 9201, CCY 9503 and CCY 9506) PCR resulted in the production of two very faint bands (~1,000 bp and ~ 600 bp) (Supplementary Figure S5), which upon cloning and sequencing showed some homology to sequences present in *Synechococcus* and *Cyanobium*, indicating that the primers are not fully specific for heterocytous cyanobacteria. Nonetheless, this unspecific and inefficient amplification using the designed primers can be discerned by the size of the PCR product and ultimately by sequencing the amplified products (Supplementary Tables S9, S10).

To check if the selected primer pair (Fw1 mix B + Rv1) actually amplified *hglT*, the most abundant PCR fragments obtained from the tested cyanobacterial cultures (shown as ++ in Supplementary Figure S4) were purified and sequenced. Initially, to ensure that all the PCR fragments obtained corresponded to heterocytous cyanobacteria, we carried out an NCBI blastN search and confirmed that all analyzed PCR fragments corresponded to HG-producing cyanobacterial strains (Supplementary Table S9). However, the genera (or species) of the hit with the highest bit-score did not always correspond to genera assigned to the tested (query) strain.

**Figure 5:**
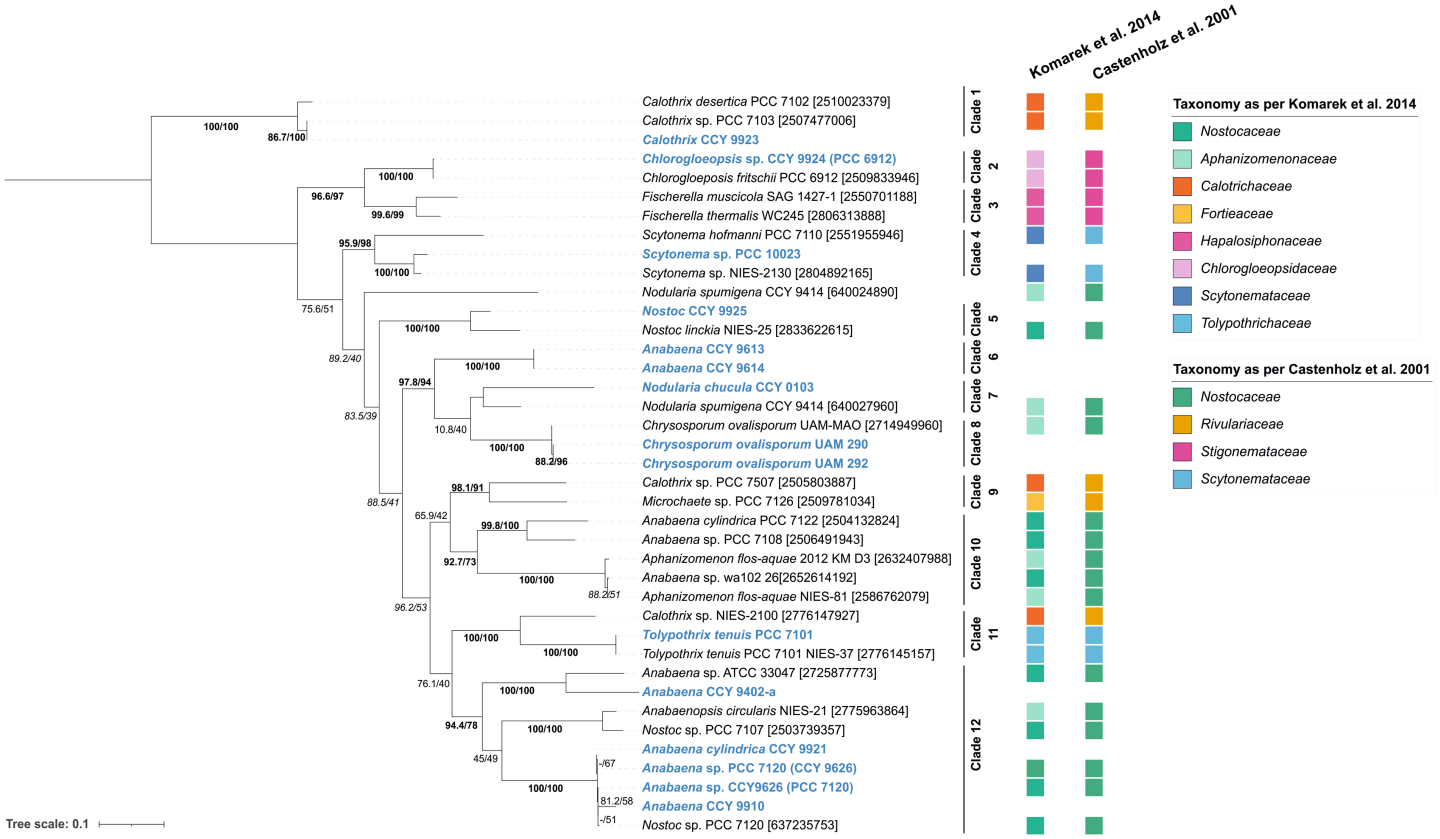
Tree containing the *hglT* gene fragments obtained via PCR and direct sequencing using primer set 2 (Fw1 mix B + Rv1) on the heterocytous cyanobacterial cultures tested in this study (sequences in blue) and *in-silico* PCR fragments obtained from the genomes used during primer design (Table 1). Tree was generated using substitution model TPM3 + F + I + G4 on IQ-tree (v1.6.7). Values on branches indicate the SH-aLRT (right side of the slash) and the standard non-parametric bootstrap supports (left side of the slash). High SH-alrt and bootstrap supports (≥85 and ≥ 70, respectively) (Hillis and Bull, 1993; Anisimova et al., 2011) are shown in bold, when only SH-alrt support is high, values are shown in italics. Gene ID is indicated between brackets. Alternative strain numbers are indicated in between parentheses. Scale bar represents the mean number of substitutions per site.

Next, we wanted to see if the amplified fragments corresponding to *hglT* could be used as phylogenetic marker and assist during strain identification and naming. To do so we generated a phylogenetic tree containing the PCR fragments (700–870 bp) obtained from the cultures analyzed in this study and PCR fragments generated *in silico* (Figure 5). *In silico* PCR fragments were obtained from the *hglT* sequences initially used to design the primers (Supplementary Table S4) by selecting the DNA fragment of *hglT* contained between primers Fw1 mixB and Rv1. This tree (Figure 5) shows that the targeted *hglT* fragment has potential as a phylogenetic marker up to the genus level. We observe that the sequences obtained using our primers, in most cases, cluster with *hglT* fragments of strains of the same genera (Figure 5). For example, *Calothrix* CCY 9923 clusters with *Calothrix* sp. PCC 7103 and *Calothrix desertica* PCC 7102 (Clade 1), confirming its affiliation to the genus *Calothrix*, while *Scytonema* sp. PCC 10023 clusters with *Scytonema* sp. NIES-2130 and *Scytonema hofmanmi* PCC 7110 (Clade 4), also confirming its affiliation to the genera *Scytonema*. Clade 12 contains closely related sequences that are identified with different names and strain identifiers, including *Anabaena* CCY 9910, *Anabaena cylindrica* CCY 9921 and three *Anabaena*/*Nostoc* sp. PCC 7120. The latter is also shown in the tree as *Anabaena* sp. CCY 9626, which refers to strain PCC 7120 after being kept in the former Culture Collection Yerseke (CCY). Strain PCC 7120 has been renamed several times, underscoring the difficulties faced by taxonomists during cyanobacterial classification. It was known first as *Nostoc muscorum*, it was then classified as *Anabaena* (Rippka et al., 1979) and later renamed as *Nostoc* although this reassignment has been questioned by some studies (Svenning et al., 2005 and references within).

Overall, these results show that *hglT*-primer set 2 is sensitive enough to detect the gene of the glycosyltransferase involved in HG biosynthesis in heterocyte-forming cyanobacteria and that upon sequencing the resulting *hglT* fragments can be used as taxonomic marker.

### Application of the developed genetic approach to the environment

After confirmation that the primer set 2 (Fw mix B and Rv1) successfully targeted *hglT* in heterocytous cyanobacterial cultures, it was tested in the environment using a microbial mat collected from the Dutch Wadden island of Schiermonnikoog. The lipid and microbial composition of microbial mats from this location had been subject of several studies in the past and were shown to contain cyanobacterial communities and heterocyte glycolipids, suggesting the presence of heterocytous cyanobacteria (Severin and Stal, 2008; Severin et al., 2010; Bauersachs et al., 2011; Fan et al., 2015).

Lipid analysis of the microbial mat confirmed the presence of heterocyte glycolipids, albeit at very low concentrations. We detected presence of 1-(O-hexose)-3,25-hexacosanediol (HG_26_ diol), 1-(O-hexose)-3-keto-25-hexacosanol (HG_26_ keto-ol), and 1-(O-hexose)-27-keto-3,25-octacosanediol (HG_28_ keto-diol), and also small amounts of 1-(O-hexose)-3,27-octacosanediol (HG_28_ diol) and 1-(O-hexose)-3,25,27-octacosanetriol (HG_28_ triol) (Table 3). Detection of HG_26_ keto-ol and diol suggest the presence of cyanobacterial members of the *Nostocaceae* family (*Nostocaceae* and *Aphanizomenonaceae*), and presence of HG_28_ keto-diol and triol suggested the presence of members of the *Rivulariaceae* (*Calotrichaceae*) family (Table 1; Supplementary Table S1). While traces of HG_28_ diol could also indicate the presence of organisms belonging to other cyanobacterial families such as *Fortiea contorta* PCC 7126 (formerly known as *Microchaete* sp.), *Stigonema ocellatum* SAG 48.90 or *Tolypothrix tenuis* PCC 7101. (Supplementary Table S1).

**Table 3:** Heterocyte glycolipids present in the microbial mat collected from Schiermonnikoog (53°29.445′N, 6°8.342′E) in April 2010.

	Relative abundance (%)
1-(O-hexose)-3,25-hexacosanediol (HG_26_ diol)	38%
1-(O-hexose)-3-keto-25-hexacosanol (HG_26_ keto-ol)	17%
1-(O-hexose)-27-keto-3,25-octacosanediol (HG_28_ keto-diol)	40%
1-(O-hexose)-3,27-octacosanediol (HG_28_ diol)	1%
1-(O-hexose)-3,25,27-octacosanetriol (HG_28_ triol)	5%

These HG results agree with the study of Bauersachs et al. (2011). These authors reported the presence of 1-(O-hexose)3,25-hexacosanediol (HG_26_ diol), suggesting the presence of members of the *Nostocaceae* family (*Nostocaceae* and *Aphanizomenonaceae*), in supratidal microbial mats collected near our sampling site. Additionally, Bauersachs et al. (2011) also detected the presence of *Calothrix* sp. and *Nodularia* sp. (*Aphanizomenaceae*) in nearby sampling sites using 16S rRNA gene libraries, although HGs generally associated with *Calothrix* were not detected.

Next, we wanted to detect potential HG-producing heterocytous cyanobacteria in the microbial mat using our newly developed *hglT* primers. DNA from the freeze-dried microbial mat was extracted as described previously for the cyanobacterial cultures (Table 2). We carried out a PCR reaction on the microbial mat DNA extract using primer pair 2 (Fw1 mixB + Rv2) and the same annealing temperature that was selected for PCR on DNA extracts of cyanobacterial cultures (56°C annealing temperature, 1′ 30″ extension time, 30 cycles). Unfortunately, however, no PCR products were obtained from the microbial mat (Supplementary Figure S6). We carried out additional tests to rule out the presence of inhibitors that could seriously hinder the reaction, and to ensure that the selected sample contained DNA belonging to cyanobacteria and nitrogen fixing bacteria (See Supplementary Results and Supplementary Figures S7–S9).

We performed a gradient PCR (48–58°C annealing temperature, 1′10″ extension time, 37 cycles) on our microbial mat sample using the *hglT*-primer mix 2 (Fw1 mixB and Rv1) and increased the number of amplification cycles to try to obtain a PCR product. However, no clear band was obtained at any of the annealing temperatures tested (Supplementary Figure S10). Previous genetic studies showed that microbial mats collected from this location contained diazotrophic cyanobacteria, and lipid analysis indicated that heterocytous cyanobacteria were present in the microbial mat (Severin et al., 2010; Bauersachs et al., 2011). However, our primers designed for the amplification of *hglT* gene fragments of heterocyte cyanobacteria did not yield any band when applied on the DNA extract of the microbial mat. This can be explained in two ways: (1) no heterocytous cyanobacteria were present in the microbial mat or they were present in very low abundances (2) our primers cannot target the heterocytous cyanobacterial species present in this microbial mat.

To answer this question, we carried out 16S rRNA gene amplicon sequencing analysis. Sequencing results showed that nearly half of the reads (54%) corresponded to Amplicon Sequence Variants (ASVs) (Table 4) of cyanobacteria. *Coleofasciculus chthonoplastes* PCC 7420, a non-heterocytous diazotroph, was the most relatively abundant (ra) cyanobacteria (43.7% ra). Other cyanobacterial species such as the nitrogen fixers *Lyngbya aestuarii* PCC-7419 (2.8% ra) and *Trichodesmium* IMS101 (0.28% ra) were also detected but no heterocytous cyanobacteria were detected (Table 4). Hence, we conclude that the amplification of *hglT* using our primers failed simply because heterocytous cyanobacteria were absent or present in very low abundances. Since the use of PCR-based approaches (including the use of universal 16S rRNA primers) typically favors amplification of more abundant microbial species, potentially masking the presence of those present in lower proportion, it is possible that the heterocytous cyanobacteria were present in the microbial mat but in too low abundances to be detected by our PCR methods. Alternatively, given the excellent preservation of heterocyte glycolipids over time (Schaefer et al., 2020), and the relatively short life of DNA when released in the environment after cell death, it is also plausible that the heterocyte glycolipids detected in the microbial mat were preserved remnants of dead heterocytous cyanobacteria whose DNA had been degraded. Therefore, both explanations would be consistent with the very low levels of HG lipids detected in the microbial mat and the absence of DNA from heterocytous cyanobacteria.

**Table 4:** Relative abundances of the cyanobacterial ASVs* present in the microbial mat and *S. fluitans* sample, obained after runing the Cascabel pipeline (Abdala Asbun et al., 2020).**

ASV ID	Microbial Mat	*Sargassum fluitans^1^*
*Acrophormium* PCC 7375	–	3.69%
Chloroplast	3.96%	–
*Chroococcidiopsis* PCC 6712	–	1.34%
*Coleofasciculaceae*	0.11%	–
*Coleofasciculus* PCC 7420	0.47%	–
*Coleofasciculus* PCC 7420 *chthonoplastes*	43.69%	–
*Cyanobacteriales*	–	0.38%
*Cyanobacteriia*	–	4.46%
*Halomicronema* TFEP1	–	0.22%
*Leptolyngbya* PCC 6406	–	0.16%
*Limnothrix*	0.26%	<0.01%
*Lyngbya* PCC 7419	0.16%	–
*Lyngbya* PCC 7419 *aestuarii*	2.77%	–
***Mastigocoleus* BC008**	**–**	**0.47%**
***Mastigocoleus* BC008 *testarum***	**–**	**11.26%**
MBIC10086	4.04%	0.03%
*Nodosilinea* PCC 7104	0.12%	–
*Nodosilineaceae*	–	0.22%
***Nostocaceae***	**–**	**1.05%**
*Oscillatoria* PCC 6304 *acumiata*	0.21%	–
*Oscillatoriaceae*	0.06%	–
*Oxyphotobacteria Incertae Sedis*	1.65%	0.09%
*Phormidesmiaceae*	–	4.71%
*Phormidesmiales*	–	0.09%
*Phormidesmis* ANT.LACV5.1	–	0.50%
*Phormidium* MBIC10003 *lucidum*	0.11%	0.04%
*Pleurocapsa* PCC 7319	–	0.57%
***Rivularia* PCC 7116**	**–**	**4.74%**
*Spirulina* P7	0.06%	–
*Symphothece* PCC 7002	0.09%	0.01%
*Synechococcus* CC9902	–	0.07%
*Synechococcus* PCC 7336	–	0.47%
*Trichodesmium* IMS101	0.28%	<0.01%
*Vampirovibrionaceae*	–	0.08%
Total % of cyanobacteria and chloroplasts	58.08%	34.87%

*Only ASVs with relative abundances ≥0.05 in at least one of the samples are included.

**Cyanobacteria known to synthesize heterocyte glycolipids are shown in bold.

^1^Theirlynck et al. (2023).

Next, we used a specimen of the floating macroalgae *S. fluitans* collected in the Central Atlantic Ocean. Its microbiome consists of 35.9% (ra) cyanobacteria, including several heterocytous cyanobacteria such as *Mastigocoleus* (11.7% ra) and *Rivularia* PCC 7116 (4.74% ra) (Table 4; Theirlynck et al., 2023), making it a good candidate to test our primers. Accordingly, we performed a gradient PCR (48–58°C annealing temperature, 1′10″ extension time, 37 cycles) on this specimen using the *hglT*-primer mix 2 (Fw1 mixB and Rv1). This led to successful amplification of the partial *hglT* gene when using annealing temperatures above 54.2°C. The most intense bands were observed at 54.2 and 56°C (Supplementary Figure S11), these results are in line with previous results obtained from testing the primer mix on cyanobacterial cultures and support our choice to use 56°C (instead of 58.2°C) as the preferred annealing temperature for all cyanobacterial cultures.

Thus, we have shown that the primers designed in this study can target and amplify the *hglT* gene of a wide variety of cyanobacterial species in the environment. This includes species not considered during primer design, such as *Mastigocoleous* and *Rivularia* PCC 7116, present in the microbiome of *S. fluitans*.

## Conclusion

We conclude that the primer set 2 designed in this study and consisting of an equimolar mix of seven forward primers (Fw1 mixB) and five reverse primers (Rv1) can successfully target and partially amplify the *hglT* gene of heterocytous cyanobacteria in cultures and the environment. However, it may also target non-heterocytous cyanobacteria such as *Synechococcus* albeit to a lesser extent. We conclude that our primers can be used qualitatively to assess the presence of heterocytous cyanobacteria. However, to ensure accurate results we suggest that (1) a temperature gradient PCR should be performed to ensure that the best annealing temperature for the sample analyzed is used, and (2) the PCR product should be sequenced to confirm the presence of the suspected heterocytous cyanobacteria in the sample.

## Data availability statement

The original contributions presented in the study are included in the article/Supplementary material. The 16S rRNA amplicon sequencing data presented in the study was deposited in the European Nucleotide Archive (ENA) repository, accession number PRJEB65747. Partial *hglT* sequences and phylogenetic trees presented in the study were deposited in the Zenodo repository, accession https://doi.org/10.5281/zenodo.8309792. Further inquiries can be directed to the corresponding author.

## Author contributions

RP: Conceptualization, Formal analysis, Investigation, Resources, Visualization, Writing – original draft, Writing – review & editing. NB: Formal analysis, Resources, Writing – review & editing. JS: Funding acquisition, Writing – review & editing. LV: Resources, Supervision, Writing – original draft, Writing – review & editing.

## Footnotes

^1^ https://img.jgi.doe.gov/
