# Supplementary material for: Developing a genetic approach to target cyanobacterial producers of heterocyte glycolipids in the environment
Source: Front Microbiol. 2023 Sep 27;14:1257040. doi: 10.3389/fmicb.2023.1257040 (PMC10569477; doi:10.3389/fmicb.2023.1257040)
Supplement: Supplementary file 2 [file Data_Sheet_1.pdf]

Supplementary results

To rule out the presence of potential inhibitors in the sample that would hinder the PCR reaction we performed a 1:2 dilution series of the microbial mat DNA extract and used it as template for two sets of PCR reactions (56 °C annealing temperature, 1' 10" extension time, 35 cycles); one was spiked with 5 ng of *Anabaena* sp. PCC 7120 and the other one was kept unspiked and was used as a control (Supplementary figure 7). We observed a slight increase in band intensity as the dilution factor increased in the samples spiked with *Anabaena* sp. PCC 7120 DNA (Supplementary figure 7, bottom), which could indicate the presence of PCR inhibitors in the DNA extracted from the microbial mat. However, since the PCR reactions were successful in all dilutions spiked with *Anabaena* sp. PCC 7120 DNA, we conclude that the presence of inhibitors in the microbial mat DNA extract is unlikely to be fully preventing the PCR reaction.

We suspected that perhaps the initial concentration of HG-synthesizing cyanobacteria in the microbial mat might have been too low. To tackle this issue, we could use two approaches: (1) increase the amount of template DNA or (2) increase the number of amplification cycles. Given the results observed in Supplementary figure 7, where undiluted DNA extracts resulted in slightly less PCR product than its diluted counterparts, we decided not to increase the volume of template DNA used, and instead increased the number of amplification cycles (56 °C annealing temperature, 1' 10" extension time, 37 cycles). However, this also did not result in successful amplification (Supplementary figure 6b). At this point, even though the presence of cyanobacteria and diazotrophs in microbial mats collected nearby had already been established in previous studies (Bauersachs et al., 2011; Fan et al., 2015), we decided to double check and confirm their presence also in our microbial mat by using alternative primers. We chose primers designed to preferentially amplify the *nifH* gene of cyanobacteria (Olson et al., 1998), and the 16S rRNA gene from cyanobacteria and chloroplasts (Nübel et al., 1997).

Amplification of *nifH* was successful (50 °C annealing temperature, 30" extension time, 37 cycles) (Supplementary figure 8) but resulted in bands slightly larger (~ 380 bp) than expected (~ 325 bp as described in Olson Olson et al., 1998). Hence, we confirmed the presence of diazotrophs in the microbial mat. However, even though these primers were designed to have a preference towards cyanobacterial diazotrophs, they are known to also amplify the *nifH* gene of other diazotrophic organisms (Olson et al., 1998). Next, we assessed the presence of cyanobacteria in our samples using primers specifically designed to amplify 16S rRNA gene fragments from cyanobacteria and chloroplasts (Nübel et al., 1997), which lead to amplification suggesting the presence of cyanobacteria in the sample (see Supplementary figure 9 for details).

Supplementary figures

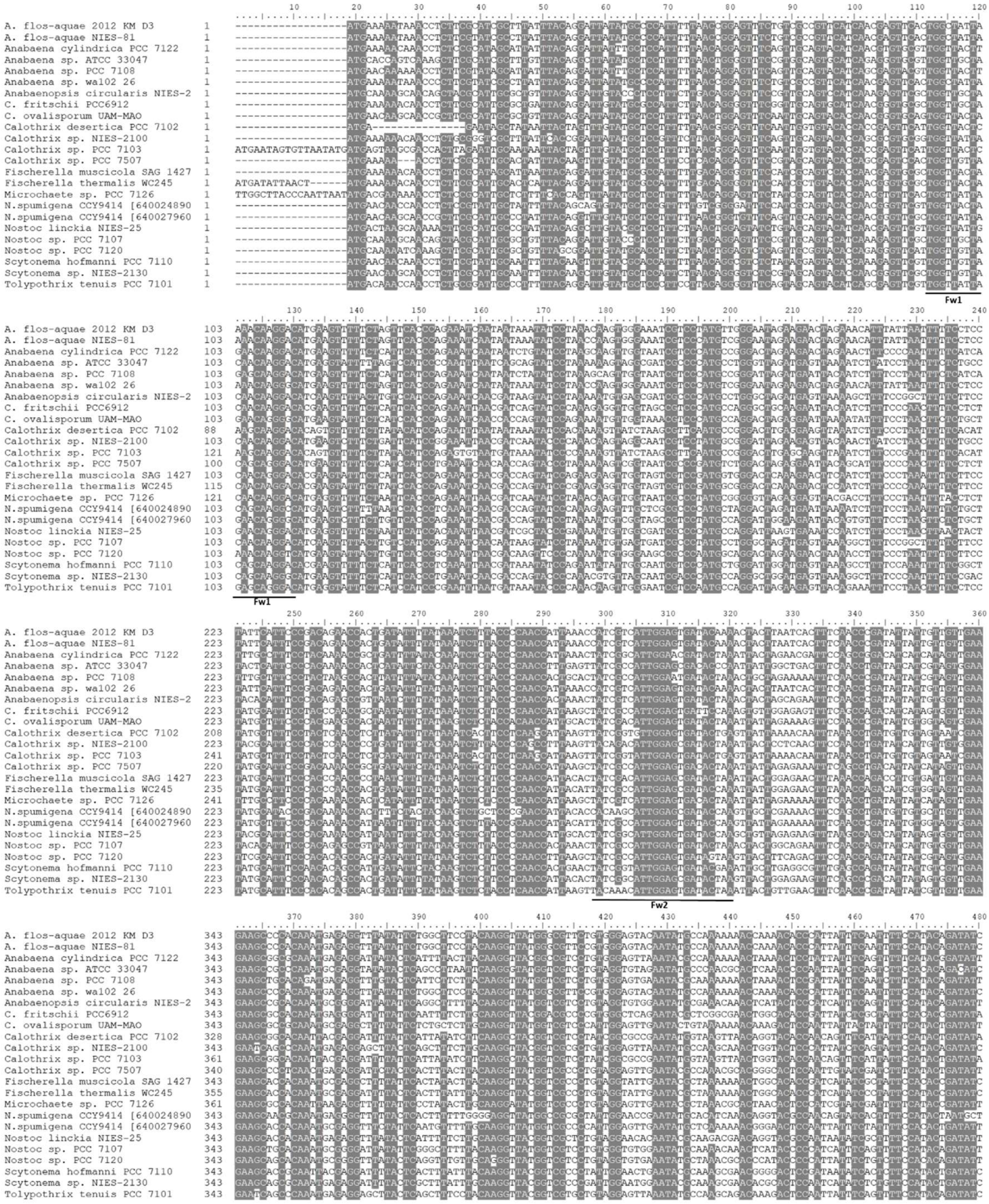

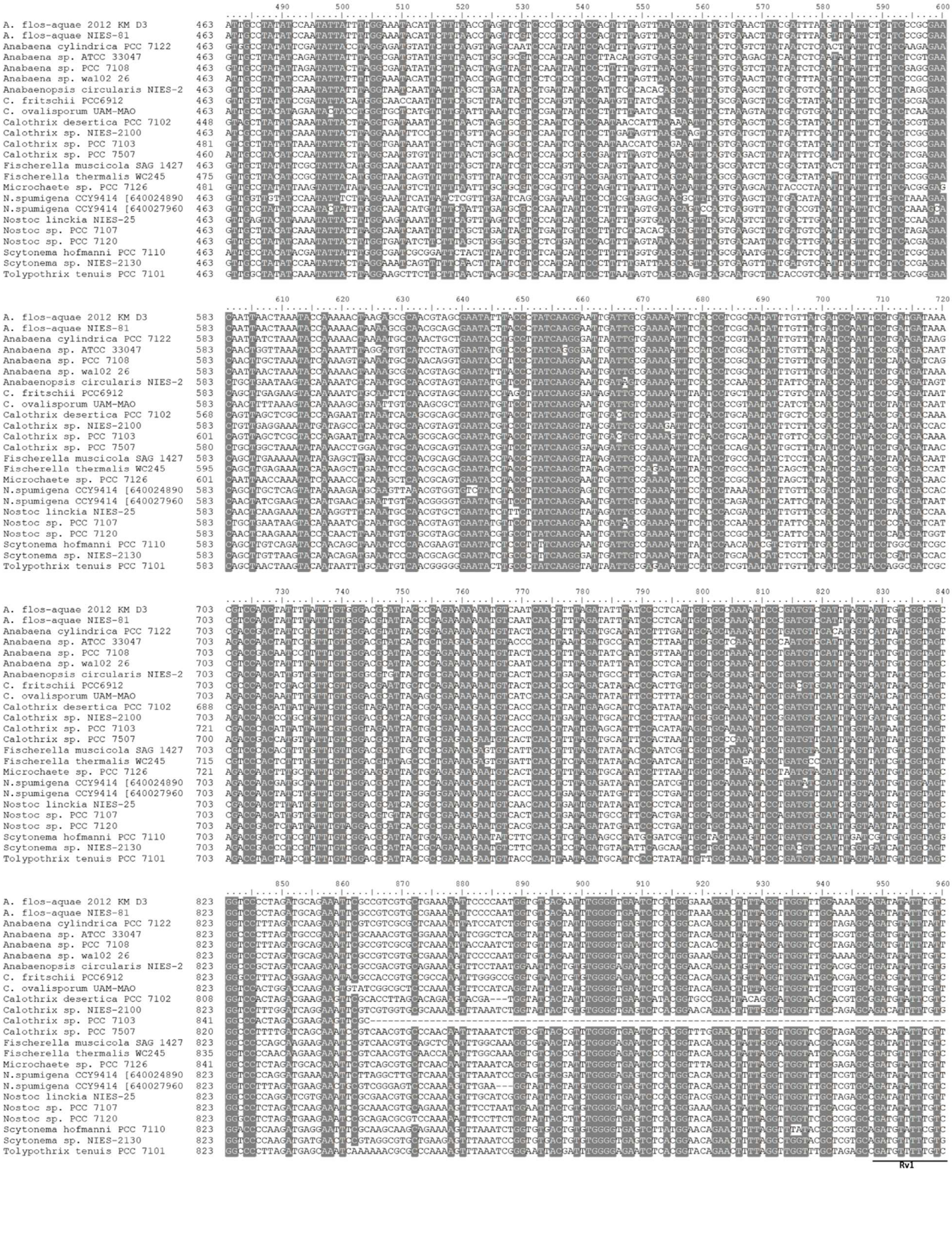

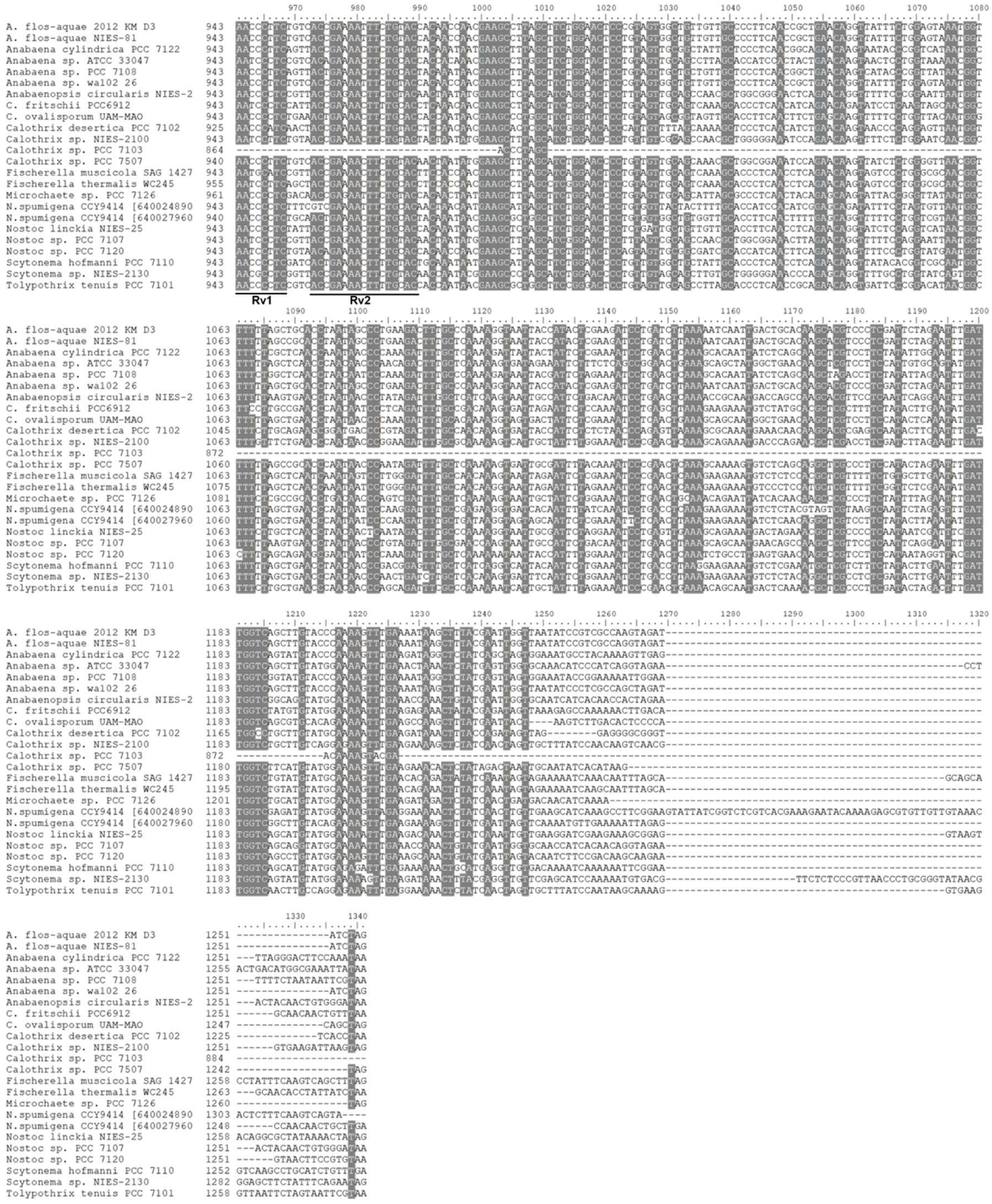

Supplementary figure 1. hglT gene sequence alignment (MUSCLE) used to identify regions suitable for primer design according to the guidelines described in the material and methods section. Selected binding regions are underlined. Background shading indicates conserved base pairs (≥ 90%).

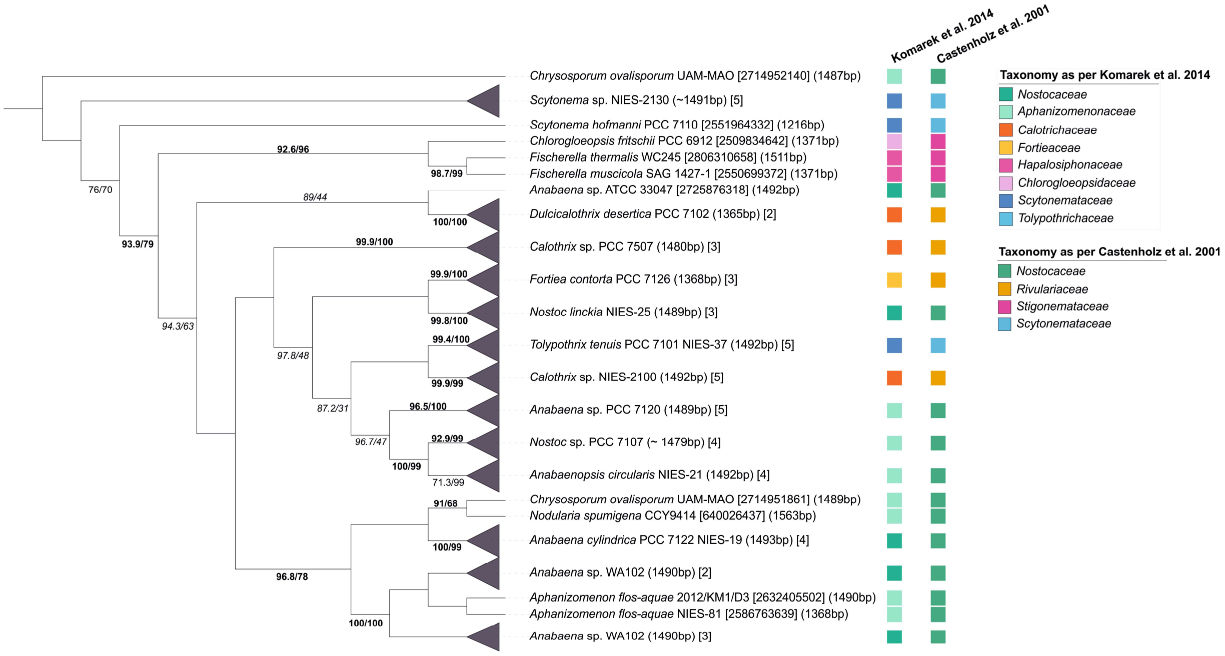

Supplementary figure 2. Cladogram of the 16S rRNA tree shown in Figure 3b. Values on branches indicate the SH-aLRT (left side of the slash) and the standard non-parametric bootstrap supports (right side of the slash). High SH-alrt and bootstrap supports (≥85 and ≥70, respectively) (Anisimova et al., 2011; Hillis & Bull, 1993) are shown in bold, when only SH-alrt support is high, values are shown in italics.

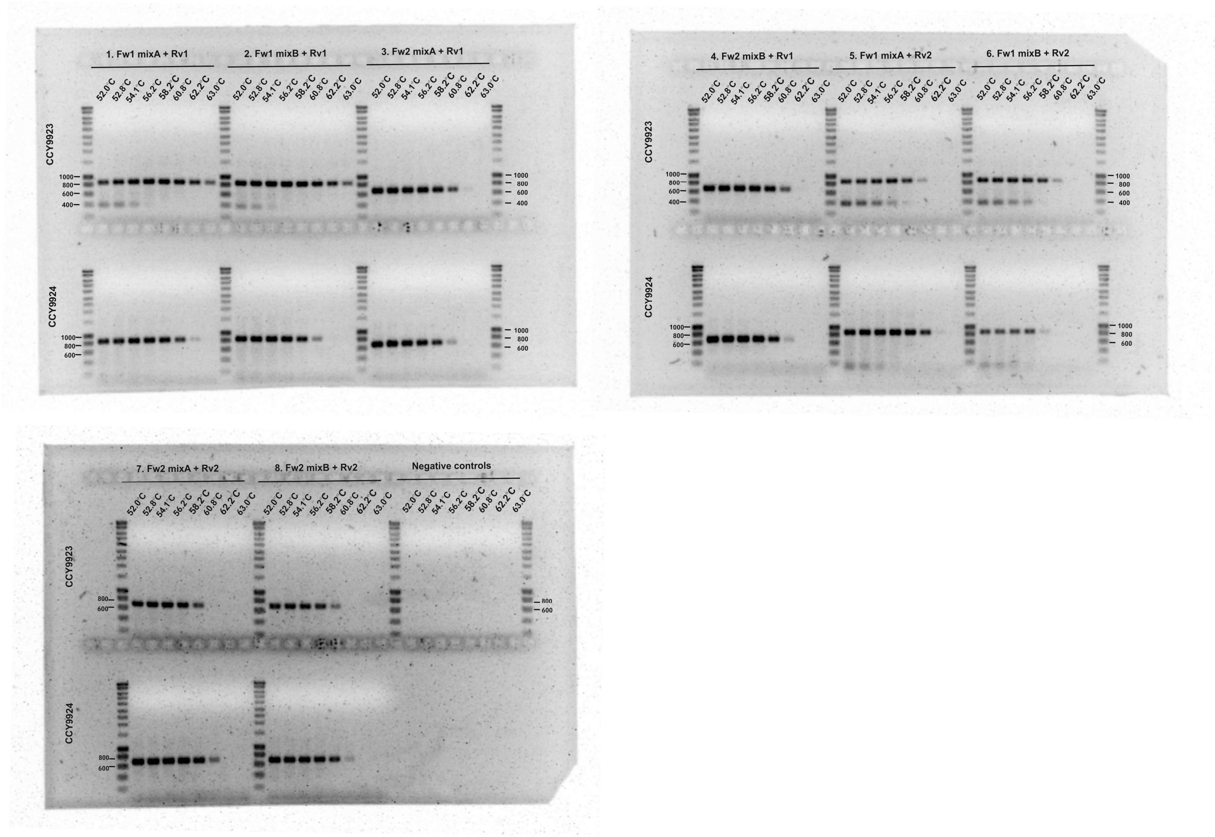

Supplementary figure 3. 1% Agarose gel (1%) stained with ethidium bromide (EtBr) showing the PCR products obtained after PCR (1'20" extension time, 30 cycles) using a range of annealing temperatures (52 ℃ to 63℃) for each of the primer combinations (1 to 8) on DNA extracts of two cyanobacterial strains: Calothrix CCY9923 (top lanes of each gel) and Chlorogloeopsis sp. CCY9924 (also known as PCC 6912) (bottom lanes of each gel). PCR water was used instead of DNA in negative controls. Annealing temperatures of 56.2 ℃ and 58.2 ℃ both seem good options, they are not very permissive and yield only one band for nearly all primer combinations in both test strains. However, in some cases less PCR product was obtained when setting the annealing temperature at 58.2 ℃, e.g., when using primers Fw2 mix B and Rv2 on CCY 9923.

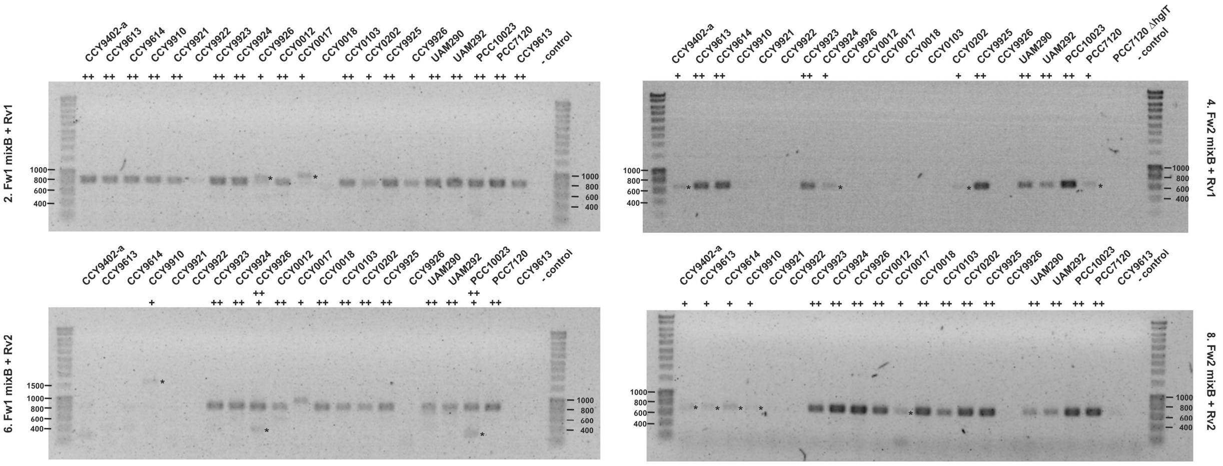

*Supplementary figure 4. Agarose gel (1%) stained with EtBr revealing the PCR products obtained after PCR (56 ℃ annealing temperature, 1'20" extension time, 30 cycles) using primers Fw1 mix B and Fw2 mix B in combination with Rv1 and Rv2 (primer combinations 2, 4, 6 and 8) with extracted DNA of 20 strains of heterocytous cyanobacteria (see Table 2). Primer set 2 (Fw1 mix B and Rv1) successfully amplified the hglT gene of 19 out of 21 tested strains; for two strains (Anabaena CCY 0017 and Nostoc CCY 9926) the amount of PCR product obtained was low Primer pair 4 (Fw2 mix B + Rv1) only amplified 11 strains (4 of them very weakly), pair 6 (Fw1 mix B + Rv2) amplified 14 strains (2 of them very weakly) and pair 8 amplified 17 strains (5 of them very weakly). Most of the PCR products were of their expected size (Supplementary figure 3). However, Anabaena strain CCY 0017 shows PCR fragments slightly larger (~1000 bp) than the rest of the strains when using primer Fw1 mix B (Supplementary figure 3). Additionally, the use of primer pair 6 (Fw2 mixB + Rv2) on strain Anabaena CCY 9910 results in a ~1600 bp band, which is significantly larger than expected (887 bp). And the same primer pair in Nostoc CCY 9926 and Scytonema sp. PCC 10023 produces an additional ~400 bp band. In wells labelled as negative controls (-control) PCR water was used instead of DNA. For primer pair 4 (Fw2 mixB + Rv1) a mutant strain of Anabaena sp. PCC 7120 lacking the hglT (ΔhglT) was also used as negative control. Key: strength of the band: ++ intense, + faint, * location of faint bands. Tolypothrix tenuis PCC 7101 was not included in this analysis.*

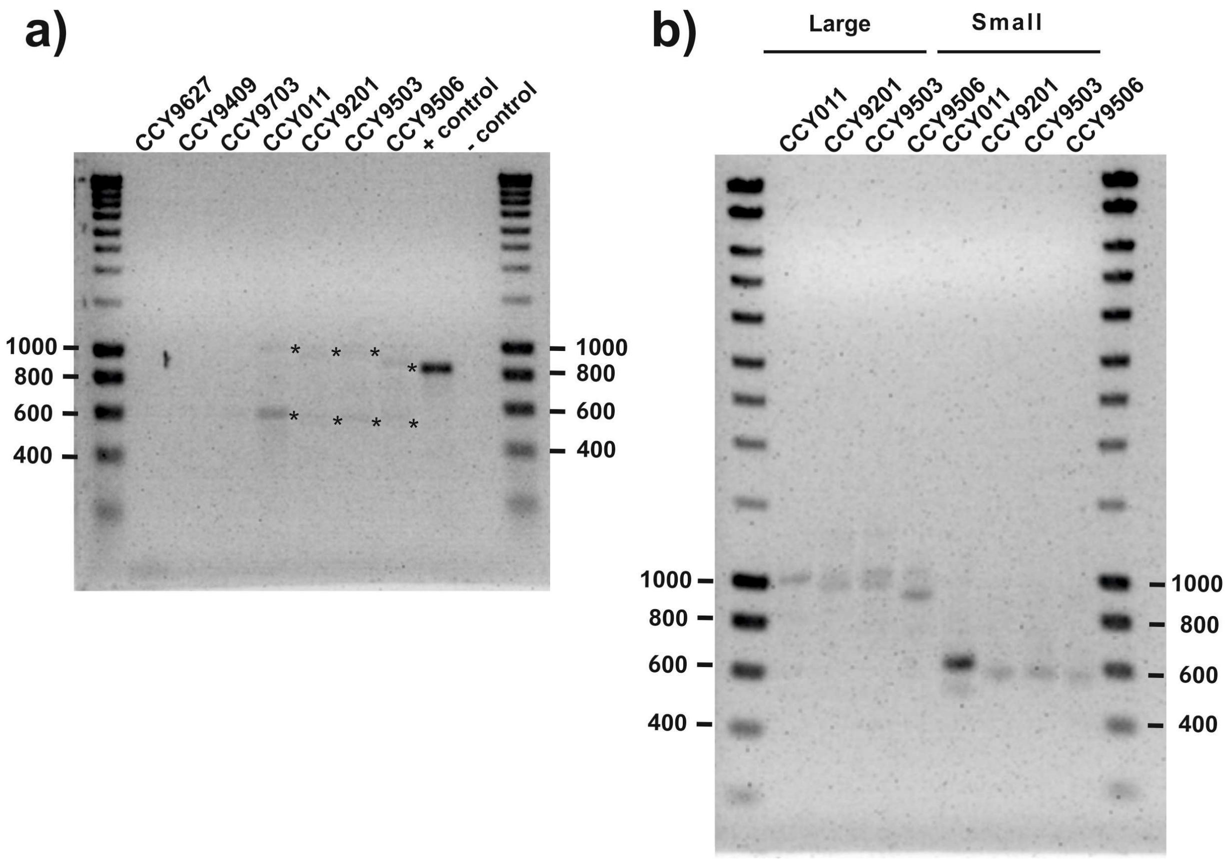

Supplementary figure 5. Agarose gel (1%) stained with EtBr showing the PCR products of four individual PCR reactions (56 °C annealing temperature, 1' extension time, 30 cycles) using primer set 2 (Fw1 mix B and Rv1) and DNA extracts of four non-heterocytous cyanobacteria (see Table 5 for strain details), before (a) and after (b) gel-purification and concentration using QIAquick gel extraction kit. Following gel purification fragments were cloned into the pCR™4-TOPO™ vector, using the TOPO-TA cloning kit and sequenced using primers T3 and T7 (Supplementary table 2).

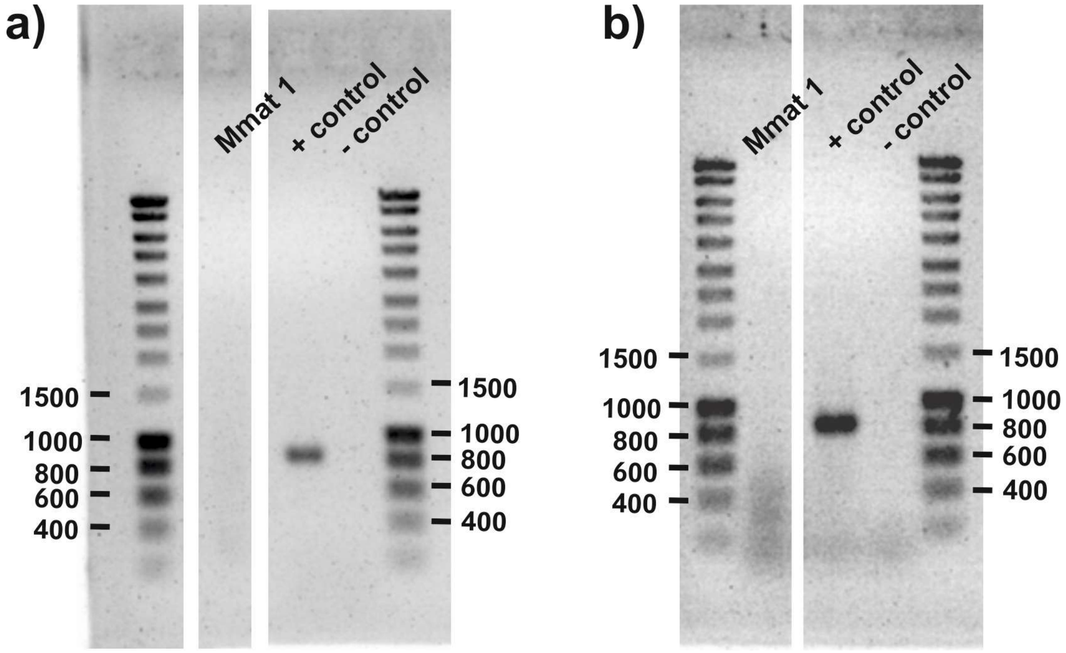

Supplementary figure 6. Agarose gel (1%) stained with EtBr showing the PCR products obtained after PCR (56 ℃ annealing temperature, 1'10" extension time, (a) 30 cycles and (b) 37 cycles) using primer set 2 (Fw1 mix B and Rv1) and DNA extracted from a microbial mat. Genomic DNA of Anabaena sp. PCC 7120 was used as positive control, and PCR water was used as negative control.

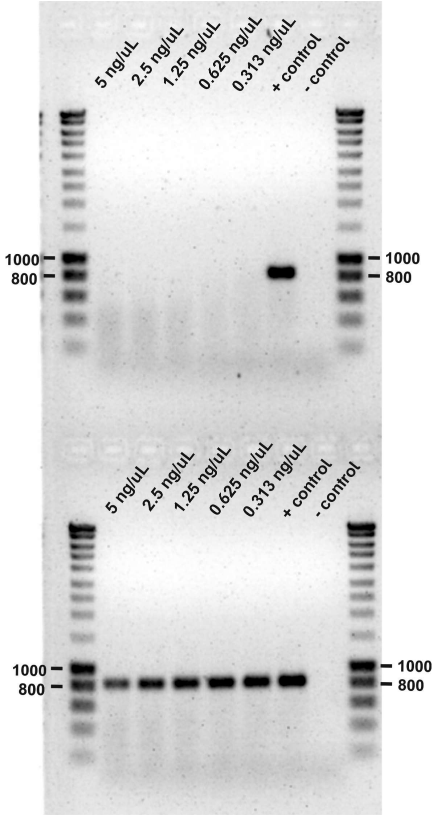

Supplementary figure 7. Agarose gel (1%) containing the product of several PCR reactions (56 ℃ annealing temperature, 1' 10" extension time, 35 cycles) using different amounts of DNA (decreasing concentration from left to right) extracted from a microbial mat with (bottom wells) and without (top wells) the addition of Anabaena sp. PCC 7120 genomic DNA (also used as positive control), to detect the potential presence of PCR inhibitors in the microbial mat DNA extract.

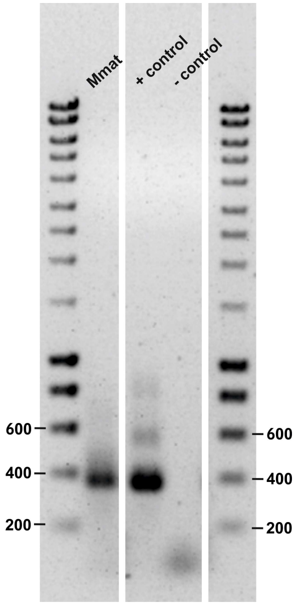

Supplementary figure 8. Agarose gel (1%) showing the PCR products obtained after PCR (50 ℃ annealing temperature, 30" extension time, 37 cycles) using primers nifHF and nifHR and DNA extracted from a microbial mat. Genomic DNA of Anabaena sp. PCC 7120 was used as positive control, and PCR water was used as negative control.

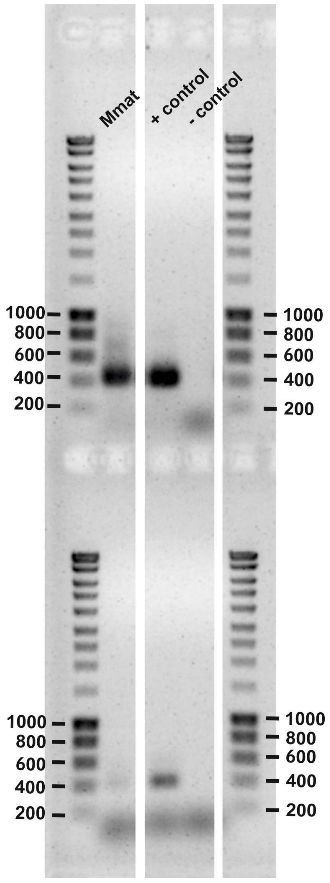

Supplementary figure 9. Agarose gel (1%) showing the PCR products obtained after PCR (50 ℃ annealing temperature, 30" extension time, 37 cycles) using DNA extracted from a microbial mat and primers CYA359F plus CYA784R (top) and CYA359F plus CYA781R (bottom) (Supplementary table 2). Genomic DNA of Anabaena sp. PCC 7120 was used as positive control, and PCR water was used as negative control. CYA781R (25 bp length) (bottom), consists of an equimolar concentration of two primers: CYA781R(a) plus CYA781R(b) (Nübel et al., 1997), which only differ in the last 3 bp.

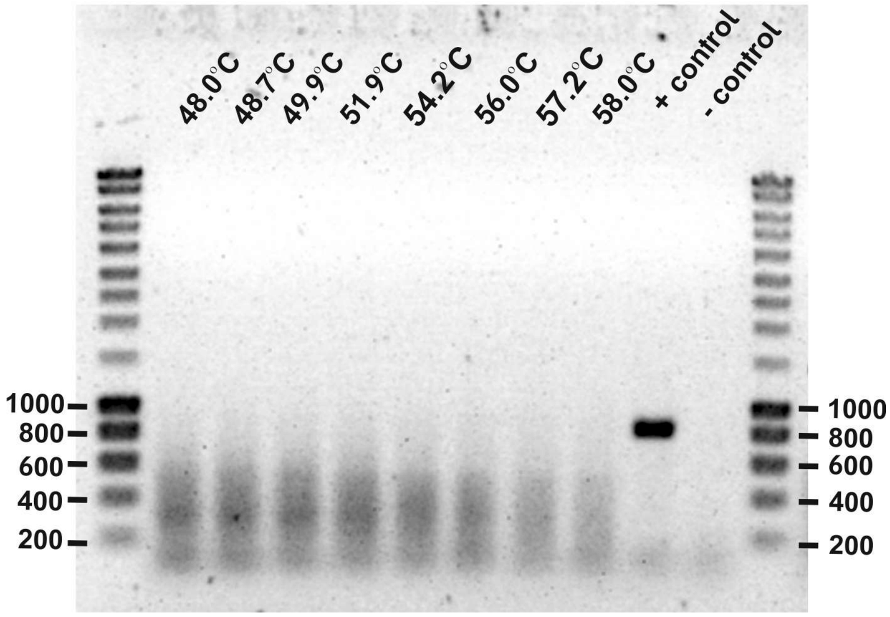

Supplementary figure 10. Agarose gel (1%) stained with EtBr showing the PCR products obtained after PCR (1'10" extension time, 37 cycles) using a range of annealing temperatures (48 ℃ to 58 ℃), primer set 2 (Fw1 mix B and Rv1) and DNA extracted from a microbial mat. Genomic DNA of Anabaena sp. PCC 7120 was used as positive control, and PCR water was used as negative control.

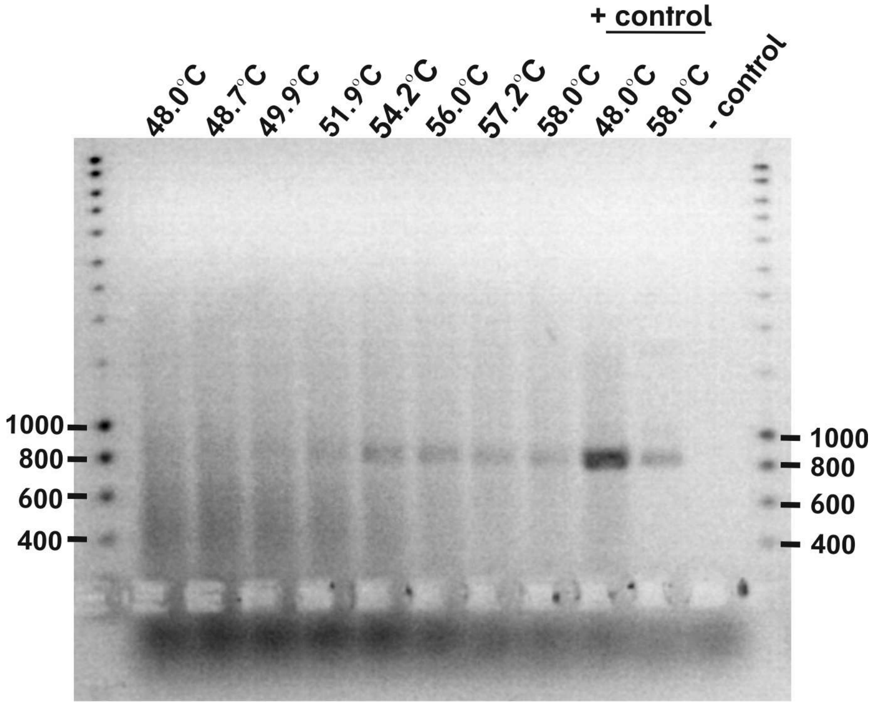

Supplementary figure 11. Agarose gel (1%) stained with GelRed® showing the PCR products obtained after PCR (1'10" extension time, 37 cycles) using a range of annealing temperatures (48 ℃ to 58 ℃), primer set 2 (Fw1 mix B and Rv1) and DNA extracted from S. fluitans. Genomic DNA of Anabaena sp. PCC 7120 was used as positive control using two annealing temperatures (48 ℃ and 58 ℃), and PCR water was used as negative control.
